# Discovery of novel dual inhibitors of receptor tyrosine kinases EGFR and PDGFR-β related to anticancer drug resistance

**DOI:** 10.1080/14756366.2017.1370583

**Published:** 2017-11-03

**Authors:** Tim Fischer, Abdulkarim Najjar, Frank Totzke, Christoph Schächtele, Wolfgang Sippl, Christoph Ritter, Andreas Hilgeroth

**Affiliations:** a Department of Pharmaceutical Chemistry, Institute of Pharmacy, Martin Luther University, Halle, Germany;; b ProQinase GmbH Freiburg, Freiburg, Germany;; c Department of Clinical Pharmacy, Institute of Pharmacy, University of Greifswald, Greifswald, Germany

**Keywords:** Benzo-anellated compounds, protein kinase inhibitory activity, biological activity

## Abstract

With ongoing resistance problems against the marketed EGFR inhibitors having a quinazoline core scaffold there is a need for the development of novel inhibitors having a modified scaffold and, thus, expected lower EGFR resistance problems. An additional problem concerning EGFR inhibitor resistance is an observed heterodimerization of EGFR with PDGFR-β that neutralises the sole inhibitor activity towards EGFR. We developed novel pyrimido[4,5-*b*]indoles with varied substitution patterns at the 4-anilino residue to evaluate their EGFR and PDGFR-β inhibiting properties. We identified dual inhibitors of both EGFR and PDGFR-β in the nanomolar range which have been initially screened in cancer cell lines to prove a benefit of both EGFR and PDGFR-β inhibition.

## Introduction

Insight in deregulated processes of cancer cells allowed the identification of novel target structures for a perspective anticancer therapy with less suspected side effects due to a more specific targeted therapy^1^. So various protein kinases have been identified as promising target structures which were found overactive in such deregulated cancer cells^2^. Being mostly overexpressed they contribute to uncontrolled cell growth or to the suppression of apoptosis^3^. Early small molecule inhibitors have been identified to inhibit the protein kinase activity in a competitive manner by binding to the ATP binding site of the respective protein kinase^4^. In order to minimise prospected side effects the first inhibitors were expected to selectively act with a single protein kinase^5^. With a specific binding mode to the protein backbone the inhibitors were found sensitive to mutations of the gene that encodes the protein to cause amino acid changes, so that certain inhibitors lost their effectiveness^6^. Alternatively, monoclonal antibodies have been developed which block the extracellular ligand binding site of the protein kinase^7^. With enormous costs of such antibody therapies there is still a need to develop novel small molecule inhibitors with alternative binding modes to the protein backbone and, thus, a lowered resistance development^7^^,^^8^.

One of the most prominent protein kinases is the endothelial growth factor receptor (EGFR) because that kinase is known to be involved in the various cancer-associated processes of uncontrolled cell growth, suppressed apoptosis, and, finally, metastasis^9^. Some mutations and resulting amino acid changes of EGFR are known to be tolerated by the inhibitors, so that the inhibitors maintain their activities, whereas, other mutations cause a loss of the inhibitory activity^10^. So recent research developments concentrated on developing alternative EGFR inhibitors^11^.

However, novel inhibitors of EGFR are suspected to suffer from similar anticancer drug resistances than the established inhibitors. Beside so far unknown mutations which may affect the inhibitor binding to EGFR, another mechanism of drug resistance may lower the inhibitor activity: a receptor heterodimerization^12^. Normally, the ligand-activated EGFR undergoes a receptor dimerisation with a neighboured EGFR receptor^13^. That dimerisation causes conformational changes that allow an autophosphorylation of tyrosine residues of the receptor dimer itself. Those phosphate groups are transferred to the intracellular EGFR substrates which mediate the further cellular signalling^14^. However, it is known that EGFR may also undergo a heterodimerization with the related platelet derived growth factor receptor (PDGFR)-β^15^^,^^16^. That receptor is found in many types of cancer similar to EGFR^1^. A dual inhibition of both EGFR and PDGFR-β would be of favour to prevent a kinase inhibitor resistance caused by a receptor heterodimerization. Recent research development concentrated on the inhibition of only PDGFR-β without affecting EGFR^17^.

We developed novel pyrimido[4,5-*b*]indoles which promise an alternative EGFR binding mode due to the additional indole nitrogen next to the *N*1 of the pyrimidine. The indole phenyl ring with its substitution and the 2-amino function of the molecular scaffold may support a target structure bonding. We varied the substitution patterns of the 4-anilino residue and determined the affinity to EGFR and PDGFR-β in an ATP competition assay using radioactively marked ATP which is incorporated into the kinase substrate and measured using scintillation counting technique.

## Experimental

### General

Commercial reagents were used without further purification. The 4-chloro pyrimidine **1b** was prepared according to literature starting from the prepared 6-amino-2-mercatopyrimidine-4-ol given by the reaction of cyano acetic acid methyl ester and thiourea in sodium methanolate, a following thioether hydrolysis using raney nickel and a final 4-chlorination reaction using phosphorus oxychloride in DMF^18^. The ^1^H-NMR spectra (400 MHz) were measured using tetramethylsilane as internal standard. TLC was performed on E. Merck 5554 silica gel plates. The ESI spectra were recorded on a Finnigan LCQ Classic mass spectrometer. Elemental analysis indicated by the symbols of the elements was within ±0.4% of the theoretical values and was performed using a Leco CHNS-932 apparatus.

### Formation of the N^4^ -anilino substituted pyrimidines 2

**Scheme 1.**
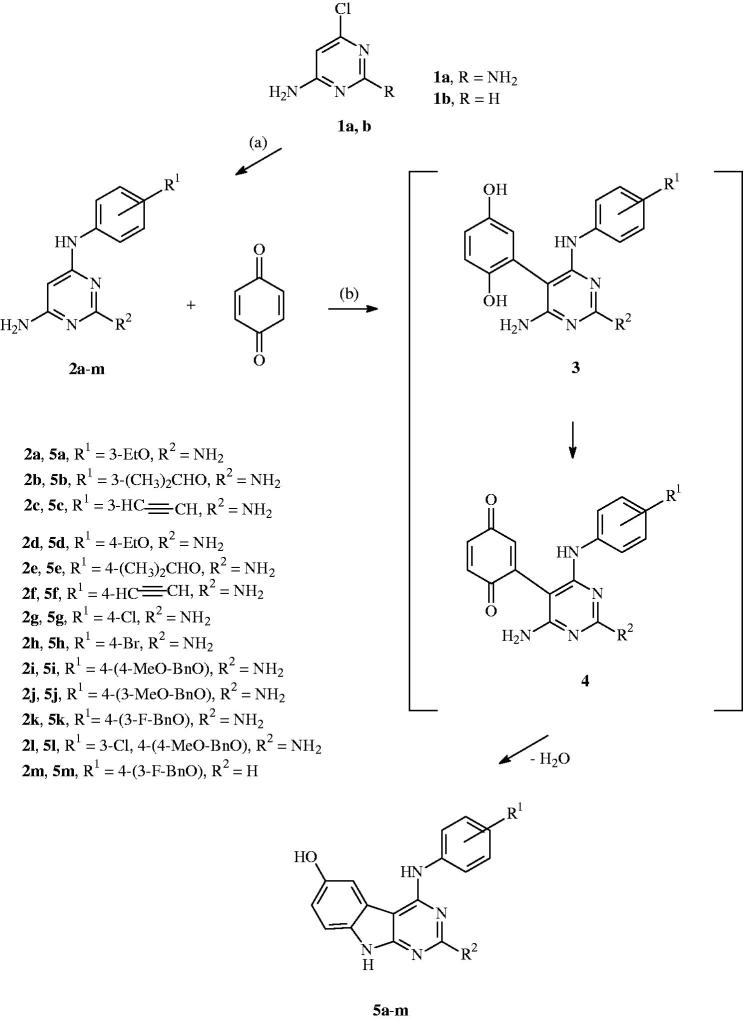
Formation of the 4-anilino substituted pyrimidines **2** and the 4-anilino substituted target compounds **5**: (a) 2–4 h, 135 °C; (b) 3–6 h, reflux, EtOH, and AcOH.

One Equivalent of the 4-chloropyrimidine **1** was mixed with three equivalents of the aniline compound except of the formation of compound **2f** and of **2h** (Scheme 1). For those reactions 1.5 equivalents (**2f**) and 4 equivalents of the aniline (**2h**) were used. In case of the formation of products **2c**, **2f**, and **2h** 1 ml of *N*-methyl pyrrolidone was additionally added. The reaction mixture was heated to 135 °C and maintained at that temperature for 2–4 h depending on the reaction proceeding followed by tlc until no more of the pyrimidine starting compound was detectable. After cooling to room temperature the raw product was purified by column chromatography using silica gel and a mixture of ethyl acetate and methanol 95/5 to 90/10 in a gradient elution. The isolated yellow, oily products were partly crystallised from methanol/diethyl ether mixtures.

*N^4^–(3-ethoxyphenyl)pyrimidine-2,4,6-triamine****2a*** Yield 1.89 g (77%); yellow oil; ^1^H NMR (DMSO-d_6_) *δ* 1.30 (t, *J* = 7.03 Hz, 3H, OCH_2_C**H**_3_), 3.98 (q, *J* = 7.0 Hz, 2H, OC**H**_2_CH_3_), 5.27 (s, 1H, 5-H), 6.33 (br, 2H, C_2_-NH_2_), 6.40 (br, 2H, C_6_-NH_2_), 6.50 (ddd, *J* = 7.99 Hz, 2.00, 1.0 Hz, 1H, 4′-H), 7.04 (ddd, *J* = 7.99, 2.00, 1.0 Hz, 1H, 6′-H), 7.11 (t, *J* = 7.99 Hz, 1H, 5′-H), 7.16 (t, *J* = 2.00 Hz, 1H, 2′-H), 8.90 (br, 1H, NH-Ph); MS (ESI), *m/z* = 246 [M + H^+^].

*N^4^–(3-isopropoxyphenyl)pyrimidine-2,4,6-triamine****2b*** Yield 2.01 g (76%); yellow oil; ^1^H NMR (DMSO-d_6_) *δ* 1.24 (d, *J* = 5.96 Hz, 6H, OCH(C**H**_3_)_2_), 4.54 (hep, *J* = 5.96 Hz, 1H, OC**H**(CH_3_)_2_), 5.29 (s, 1H, 5-H), 6.49–6.51 (m, 3H, 4′-H, C_2_-NH_2_), 6.56 (br, 2H, C_6_-NH_2_), 7.04–7.11 (m, 3H, 2′-, 5′-, 6′-H), 8.90 (br, 1H, NH-Ph); MS (ESI), *m/z* = 260 [M + H^+^].

*N^4^–(3-ethinylphenyl)pyrimidine-2,4,6-triamine****2c*** Yield 0.63 g (56%); yellow oil; ^1^H NMR (DMSO-d_6_) *δ* 4.10 (s, 1H, CH≡C), 5.21 (s, 1H, 5-H), 5.88 (br, 2H, C_2_-NH_2_), 6.03 (br, 2H, C_6_-NH_2_), 6.97 (dt, *J* = 7.60 Hz, 1.25 Hz, 1H, 4′-H), 7.21 (t, *J* = 7.60 Hz, 1H, 5′-H), 7.62 (t, *J* = 1.25 Hz, 1H, 2′-H), 7.04 (dt, *J* = 7.60, 1.25 Hz, 1H, 6′-H), 8.72 (br, 1H, NH-Ph); MS (ESI), *m/z* = 226 [M + H^+^].

*N^4^–(4-ethoxyphenyl)pyrimidine-2,4,6-triamine****2d*** Yield 1.62 g (66%); yellow oil; ^1^H NMR (DMSO-d_6_) *δ* 1.29 (t, *J* = 6.92 Hz, 3H, OCH_2_C**H**_3_), 3.95 (q, *J* = 6.92 Hz, 2H, OC**H**_2_CH_3_), 5.11 (s, 1H, 5-H), 5.85 (br, 2H, C2-NH_2_), 5.98 (br, 2H, C_6_-NH_2_), 6.80 (AA′**BB′**, 2H, 3′-, 5′-H), 7.35 (**AA′**BB′, 2H, 2′, 6′-H), 8.39 (br, 1H, NH-Ph); MS (ESI), *m/z* = 246 [M + H^+^].

*N^4^–(4-isopropoxyphenyl)pyrimidine-2,4,6-triamine****2e*** Yield 1.88 g (73%); yellow oil; ^1^H NMR (DMSO-d_6_) *δ* 1.25 (d, *J* = 6.02 Hz, 6H, OCH(C**H**_3_)_2_), 4.56 (hep, *J* = 6.02 Hz, 2H, OC**H**(CH_3_)_2_), 5.13 (s, 1H, 5-H), 5.92 (br, 2H, C_2_-NH_2_), 6.03 (br, 2H, C_6_-NH_2_), 6.83 (AA′**BB′**, 2H, 3′-, 5′-H), 7.36 (**AA′**BB′, 2H, 2′, 6′-H), 8.50 (br, 1H, NH-Ph); MS (ESI), *m/z* = 260 [M + H^+^].

*N^4^–(4-ethinylphenyl)pyrimidine-2,4,6-triamine****2f*** Yield 0.59 g (52%); yellow oil; ^1^H NMR (DMSO-d_6_) *δ* 2.82 (s, 1H, CH≡C), 5.17 (s, 1H, 5-H), 5.64 (br, 2H, C_2_-NH_2_), 5.82 (br, 2H, C_6_-NH_2_), 7.33 (AA′**BB′**, 2H, 3′-, 5′-H), 7.60 (**AA′**BB′, 2H, 2′, 6′-H), 8.63 (br, 1H, NH-Ph); MS (ESI), *m/z* = 226 [M + H^+^].

*N^4^–(4-chlorophenyl)pyrimidine-2,4,6-triamine****2g*** Yield 1.43 g (61%); mp 145–147 °C; ^1^H NMR (DMSO-d_6_) *δ* 5.38 (s, 1H, 5-H), 4.82 (br, 4H, C_2_-NH_2_, C_6_-NH_2_), 7.32 (AA′BB′, 4H, 2′-, 6′-, 3′-, 5′-H), 7.50 (br, 1H, NH-Ph); MS (ESI), *m/z* = 236 [M + H^+^].

*N^4^–(4-bromophenyl)pyrimidine-2,4,6-triamine****2h*** Yield 1.13 g (81%); yellow oil; ^1^H NMR (DMSO-d_6_) *δ* 5.20 (s, 1H, 5-H), 5.95 (br, 2H, C_2_-NH_2_), 6.07 (br, 2H, C_6_-NH_2_), 7.36 (AA′**BB′**, 2H, 3′-, 5′-H), 7.59 (**AA′**BB′, 2H, 2′, 6′-H), 8.82 (br, 1H, NH-Ph); MS (ESI), *m/z* = 280 [M + H^+^].

*N^4^–(4-((4-methoxybenzyl)oxy)phenyl)pyrimidine-2,4,6-triamine****2i*** Yield 2.66 g (79%); mp 179–181 °C; ^1^H NMR (DMSO-d_6_) *δ* 3.74 (s, 3H, OCH_3_), 4.95 (s, 2H, OCH_2_), 5.10 (s, 1H, 5-H), 5.88 (br, 2H, C_2_-NH_2_), 6.01 (br, 2H, C_6_-NH_2_), 6.88 (AA′**BB′**, 2H, 3′-, 5′-H), 6.93 (**AA′**BB′, 2H, 2′-, 6′-H), 7.35 (m, 4H, 2″-, 3″-, 5″-,6″-H), 7.35 (br, 1H, NH-Ph); MS (ESI), *m/z* = 338 [M + H^+^].

*N^4^–(4-((3-methoxybenzyl)oxy)phenyl)pyrimidine-2,4,6-triamine****2j*** Yield 2.96 g (88%); mp 172–174 °C; ^1^H NMR (DMSO-d_6_) *δ* 3.74 (s, 3H, OCH_3_), 5.02 (s, 2H, OCH_2_), 5.11 (s, 1H, 5-H), 5.93 (br, 2H, C_2_-NH_2_), 6.05 (br, 2H, C_6_-NH_2_), 6.85–6.88 (m, 1H, 4″-H), 6.89 (AA′**BB′**, 2H, 3′-, 5′-H), 6.97–7.00 (m, 2H, 2″-, 6″-H), 7.28 (t, *J* = 8.15 Hz, 1H, 5′-H), 7.37 (**AA′**BB′, 2H, 2′-, 6′-H), 8.48 (br, 1H, NH-Ph); MS (ESI), *m/z* = 338 [M + H^+^].

*N^4^–(4-((3-fluorobenzyl)oxy)phenyl)pyrimidine-2,4,6-triamine****2k*** Yield 2.58 g (79%); mp 96–98 °C; ^1^H NMR (DMSO-d_6_) *δ* 5.08 (s, 2H, OCH_2_), 5.12 (s, 1H, 5-H), 6.07 (br, 2H, C_2_-NH_2_), 6.16 (br, 2H, C_6_-NH_2_), 6.91 (AA′**BB′**, 2H, 3′-, 5′-H), 7.14 (dt, *J* = 8.65, 2.52 Hz, 1H, 4″-H), 7.23–7.28 (m, 2H, 2″-, 6″-H), 7.37 (**AA′**BB′, 2H, 2′-, 6′-H), 7.42 (dt, *J* = 8.65, 6.10 Hz, 1H, 5″-H), 8.48 (br, 1H, NH-Ph); MS (ESI), *m/z* = 326 [M + H^+^].

*N^4^–(3-chloro-4-((4-methoxybenzyl)oxy)phenyl)pyrimidine-2,4,6-triamine****2l*** Yield 2.76 g (74%); mp 140–143 °C; ^1^H NMR (DMSO-d_6_) *δ* 3.74 (s, 3H, OCH_3_), 5.06 (s, 2H, OCH_2_), 5.17 (s, 1H, 5-H), 6.66 (br, 4H, C_2_-NH_2_, C_6_-NH_2_), 6.94 (AA′**BB′**, 2H, 3″-, 5″-H), 7.15 (d, *J* = 8.98 Hz, 1H, 5′-H), 7.36 (m, 3H, 6′-, 2″-, 6″-H), 7.64 (d, *J* = 2.33 Hz, 1H, 2′-H), 8.48 (br, 1H, NH-Ph); MS (ESI), *m/z* = 372 [M + H^+^].

*N^4^–(4-((3-fluorobenzyl)oxy)phenyl)pyrimidine-4,6-triamine****2m*** Yield 1.05 g (86%); mp 184–185 °C; ^1^H NMR (DMSO-d_6_) *δ* 5.08 (s, 2H, OCH_2_), 5.65 (s, 1H, 5-H), 6.21 (br, 2H, NH_2_), 6.94 (AA′**BB′**, 2H, 3′-, 5′-H), 7.15 (m, 1H, 4″-H), 7.25–7.29 (m, 2H, 2″-, 6″-H), 7.35 (**AA′**BB′, 2H, 2′-, 6′-H), 7.43 (dt, *J* = 7.58, 6.58 Hz, 1H, 5″-H), 7.96 (br, 1H, NH-Ph), 8.61 (s, 1H, 2-H); MS (ESI), *m/z* = 311 [M + H^+^].

### Formation of the 4-anilino substituted 9H-pyrimido[4,5-b]indol-6-ols 5

One Equivalent of the *N^4^*-anilino substituted pyrimidine **2** reacted with 1.2 to 1.8 equivalents of *p*-benzoquinone in volumes of 20 ml of dried ethanol and 5 ml of dried acetic acid per 1 mmol of the used pyrimidine **2** (Scheme 1). The solution was heated under reflux for 3–6 h depending on the reaction proceeding that was followed by tlc. After reaction completion the solvent was evaporated in vacuum. The remaining oil was purified using column chromatography with silica gel and an eluent mixture of chloroform and methanol in a relation of 95 to 5 that changed gradually to a relation of 90 to 10.

*2-Amino-4-((3-ethoxyphenyl)amino)-9H-pyrimido[4,5-b]indol-6-ol****5a*** Yield 0.065 g (5%); greyish powder; mp 154–157 °C; ^1^H NMR (DMSO-d_6_) *δ* 1.33 (t, *J* = 7.0 Hz, 3H, OCH_2_C**H**_3_), 4.06 (q, *J* = 7.0 Hz, 2H, OC**H**_2_CH_3_), 5.93 (br, 2H, NH_2_), 6.57 (br, 1H, NH-Ph), 6.63 (dd, *J* = 8.57, 2.21 Hz, 1H, 7-H), 6.50 (dd, *J* = 8.18, 1.90 Hz, 1H, 4′-H), 6.96 (d, *J* = 8.57, 1H, 8-H), 7.01 (d, *J* 1.90 Hz, 1H, 2′-H), 7.05–7.04 (m, 1H, 5′-H), 7.42 (d, *J* = 8.09, 1H, 6′-H), 7.45 (d, *J* = 2.21 Hz, 1H, 5-H), 8.85 (br, 1H, OH), 11.92 (br,1H, NH); MS (ESI), *m/z* = 336 [M + H^+^]; Anal. (C_18_H_17_N_5_O_2_) Calc. C 64.47, H 5.11, N 20.88; Found C 64.07, H 5.20, N 20.55.

*2-Amino-4-((3-isopropoxyphenyl)amino)-9H-pyrimido[4,5-b]indol-6-ol****5b*** Yield 0.088 g (6%); greyish powder; mp 147–149 °C; ^1^H NMR (DMSO-d_6_) *δ* 1.29 (d, *J* = 6.0 Hz, 6H, OCH(C**H**_3_)_2_), 4.64 (hept, *J* = 6.0 Hz, 1H, OC**H**(CH_3_)_2_), 5.92 (br, 2H, NH_2_), 6.55 (br, 1H, NH-Ph), 6.61 (dd, *J* = 8.63, 2.20 Hz, 1H, 7-H), 6.93 (dd, *J* = 8.1, 1.98 Hz, 1H, 4′-H), 6.97 (d, *J* = 8.63, 1H, 8-H), 7.02 (d, *J* = 1.98 Hz, 1H, 2′-H), 7.06 (t, *J* = 8.1 Hz, 1H, 5′-H), 7.42 (d, *J* = 8.1, 1H, 6′-H), 7.46 (d, *J* = 2.2 Hz, 1H, 5-H), 8.87 (br, 1H, OH), 11.93 (br, 1H, NH); MS (ESI), *m/z* = 350 [M + H^+^]; Anal. (C_19_H_19_N_5_O_2_) Calc. C 65.32, H 5.48, N 20.04; Found C 64.95, H 5.10, N 20.32.

*2-Amino-4-((3-ethinylphenyl)amino)-9H-pyrimido[4,5-b]indol-6-ol****5c*** Yield 0.020 g (2%); greyish powder; mp 176–178 °C; ^1^H NMR (DMSO-d_6_) *δ* 4.27 (s, 1H, CH≡C), 5.97 (br, 2H, NH_2_), 6.60 (br, 1H, NH-Ph), 6.65 (dd, *J* = 8.66, 2.30 Hz, 1H, 7-H), 6.94 (d, *J* = 8.66, 1H, 8-H), 7.46 (d, *J* = 2.30 Hz, 1H, 5-H), 7.51 (ddd, *J* = 6.14, 3.01, 1.30 Hz, 1H, 6′-H), 7.55–7.58 (m, 3H, 2′-, 4′-, 5′-H), 8.89 (br, 1H, OH), 11.93 (br,1H, NH); MS (ESI), *m/z* = 316 [M + H^+^]; Anal. (C_18_H_13_N_5_O) Calc. C 68.56, H 4.16, N 22.21; Found C 68.15, H 3.85, N 22.30.

*2-Amino-4-((4-ethoxyphenyl)amino)-9H-pyrimido[4,5-b]indol-6-ol****5d*** Yield 0.096 g (7%); greyish powder; mp 255–257 °C; ^1^H NMR (DMSO-d_6_) *δ* 1.36 (t, *J* = 6.95 Hz, 3H, OCH_2_C**H**_3_), 4.09 (q, *J* = 6.95 Hz, 2H, OC**H**_2_CH_3_), 5.89 (br, 2H, NH_2_), 6.54 (br, 1H, NH-Ph), 6.61 (dd, *J* = 8.54, 2.23 Hz, 1H, 7-H), 6.83 (d, *J* = 8.54, 1H, 8-H), 7.07 (AA′**BB′**, 2H, 3′, 5′-H), 7.34 (**AA′**BB′, 2H, 2′-, 6′-H), 7.44 (d, *J* = 2.23 Hz, 1H, 5-H), 8.82 (br, 1H, OH), 11.93 (br,1H, NH); MS (ESI), *m/z* = 336 [M + H^+^]; Anal. (C_18_H_17_N_5_O_2_) Calc. C 64.47, H 5.11, N 20.88; Found C 64.12, H 5.05, N 20.48.

*2-Amino-4-((4-isopropoxyphenyl)amino)-9H-pyrimido[4,5-b]indol-6-ol****5e*** Yield 0.100 g (7%); greyish powder; mp 215–217 °C; ^1^H NMR (DMSO-d_6_) *δ* 1.31 (d, *J* = 5.97 Hz, 6H, OCH(C**H**_3_)_2_), 4.66 (hept, *J* = 5.97 Hz, 1H, OC**H**(CH_3_)_2_), 5.89 (br, 2H, NH_2_), 6.54 (br, 1H, NH-Ph), 6.61 (dd, *J* = 8.59, 2.25 Hz, 1H, 7-H), 6.84 (d, *J* = 8.59, 1H, 8-H), 7.06 (AA′**BB′**, 2H, 3′, 5′-H), 7.33 (**AA′**BB′, 2H, 2′, 6′-H), 7.44 (d, *J* = 2.25 Hz, 1H, 5-H), 8.82 (br, 1H, OH), 11.94 (br, 1H, NH); MS (ESI), *m/z* = 350 [M + H^+^]; Anal. (C_19_H_19_N_5_O_2_) Calc. C 65.32, H 5.48, N 20.04; Found C 65.15, H 5.08, N 19.85.

*2-Amino-4-((4-ethinylphenyl)amino)-9H-pyrimido[4,5-b]indol-6-ol****5f*** Yield 0.008 g (1%); greyish powder; mp >320 °C; ^1^H NMR (DMSO-d_6_) *δ* 2.63 (s, 1H, CH≡C), 5.97 (br, 2H, NH_2_), 6.63 (br, 1H, NH-Ph), 6.65 (dd, *J* = 8.70, 2.45 Hz, 1H, 7-H), 7.08 (d, *J* = 8.70, 1H, 8-H), 7.48 (d, *J* = 2.45 Hz, 1H, 5-H), 7.71 (AA′**BB′**, 2H, 3′, 5′-H), 8.12 (**AA′**BB′, 2H, 2′-, 6′-H), 8.94 (br, 1H, OH), 11.97 (br, 1H, NH); MS (ESI), *m/z* = 316 [M + H^+^]; Anal. (C_18_H_13_N_5_O) Calc. C 68.56, H 4.16, N 22.21; Found C 68.35, H 3.97, N 22.04.

*2-Amino-4-((4-chlorophenyl)amino)-9H-pyrimido[4,5-b]indol-6-ol****5g*** Yield 0.105 g (9%); greyish powder; mp 295–297 °C; ^1^H NMR (DMSO-d_6_) *δ* 5.99 (br, 2H, NH_2_), 6.63 (dd, *J* = 8.87, 2.24 Hz, 1H, 7-H), 6.66 (br, 1H, NH-Ph), 6.96 (d, *J* = 8.87, 1H, 8-H), 7.46 (d, *J* = 2.24 Hz, 1H, 5-H), 7.54 (AA′**BB′**, 2H, 3′, 5′-H), 7.61 (**AA′**BB′, 2H, 2′-, 6′-H), 8.91 (br, 1H, OH), 11.95 (br, 1H, NH); MS (ESI), *m/z* = 326 [M + H^+^]; Anal. (C_18_H_12_ClN_5_O) Calc. C 58.99, H 3.71, N 21.50; Found C 58.65, H 3.46, N 21.35.

*2-Amino-4-((4-bromophenyl)amino)-9H-pyrimido[4,5-b]indol-6-ol****5h*** Yield 0.090 g (6%); greyish powder; mp 303–305 °C; ^1^H NMR (DMSO-d_6_) *δ* 5.94 (br, 2H, NH_2_), 6.60 (br, 1H, NH-Ph), 6.64 (dd, *J* = 8.64, 2.30 Hz, 1H, 7-H), 6.97 (d, *J* = 8.64, 1H, 8-H), 7.45 (d, *J* = 2.30 Hz, 1H, 5-H), 7.48 (AA′**BB′**, 2H, 3′, 5′-H), 7.73 (**AA′**BB′, 2H, 2′-, 6′-H), 8.89 (br, 1H, OH), 11.89 (br, 1H, NH); MS (ESI), *m/z* = 370 [M + H^+^]; Anal. (C_18_H_12_BrN_5_O) Calc. C 51.91, H 3.27, N 18.92; Found C 51.75, H 3.14, N 18.55.

*2-Amino-4-((4–(4-methoxybenzyl)oxy)phenyl)amino)-9H-pyrimido[4,5-b]indol-6-ol****5i*** Yield 0.083 g (5%); greyish powder; mp 252–254 °C; ^1^H NMR (DMSO-d_6_) *δ* 3.75 (s, 3H, OCH_3_), 5.09 (s, 2H, OCH_2_), 5.90 (br, 2H, NH_2_), 6.54 (br, 1H, NH-Ph), 6.62 (dd, *J* = 8.54, 2.15 Hz, 1H, 7-H), 6.82 (d, *J* = 8.54, 1H, 8-H), 6.96 (AA′**BB′**, 2H, 3″-, 5″-H), 7.14 (AA′**BB′**, 2H, 3′-, 5′-H), 7.34 (**AA′**BB′, 2H, 2′-, 6′-H), 7.41 (**AA′**BB′, 2H, 2″-, 6″-H), 7.44 (d, *J* = 2.15 Hz, 1H, 5-H), 8.81 (br, 1H, OH), 11.92 (br, 1H, NH); MS (ESI), *m/z* = 428 [M + H^+^]; Anal. (C_24_H_21_N_5_O_3_) Calc. C 67.44, H 4.95, N 16.38; Found C 67.13, H 4.75, N 15.98.

*2-Amino-4-((4–(3-methoxybenzyl)oxy)phenyl)amino)-9H-pyrimido-[4,5-b]indol-6-ol****5j*** Yield 0.047 g (2%); greyish powder; mp 173–175 °C; ^1^H NMR (DMSO-d_6_) *δ* 3.76 (s, 3H, OCH_3_), 5.16 (s, 2H, OCH_2_), 5.91 (br, 2H, NH_2_), 6.54 (br, 1H, NH-Ph), 6.62 (dd, *J* = 8.60, 2.03 Hz, 1H, 7-H), 6.83 (d, *J* = 8.60, 1H, 8-H), 6.91 (dd, *J* = 7.75, 2.14 Hz, 1H, 4″-H), 7.04 (s, 1H, 2″-H), 7.05 (d, *J* = 7.25, 1H, 6″-H), 7.16 (AA′**BB′**, 2H, 3′-, 5′-H), 7.32 (t, *J* = 7.75 Hz, 1H, 5″-H), 7.35 (**AA′**BB′, 2H, 2′-, 6′-H), 7.44 (d, *J* = 2.03 Hz, 1H, 5-H), 8.81 (br, 1H, OH), 11.95 (br, 1H, NH); MS (ESI), *m/z* = 428 [M + H^+^]; Anal. (C_24_H_21_N_5_O_3_) Calc. C 67.44, H 4.95, N 16.38; Found C 67.14, H 5.17, N 16.24.

*2-Amino-4-((4–(3-fluorobenzyl)oxy)phenyl)amino)-9H-pyrimido[4,5-b]indol-6-ol****5k*** Yield 0.087 g (4%); greyish powder; mp 199–201 °C; ^1^H NMR (DMSO-d_6_) *δ* 5.21 (s, 2H, OCH_2_), 5.90 (br, 2H, NH_2_), 6.54 (br, 1H, NH-Ph), 6.62 (dd, *J* = 8.58, 2.29 Hz, 1H, 7-H), 6.83 (d, *J* = 8.58, 1H, 8-H), 7.15 7.19 (m, 1H, 2″-H), 7.17 (AA′**BB′**, 2H, 3′-, 5′-H), 7.31–7.35 (m, 2H, 4″-, 6″-H), 7.37 (**AA′**BB′, 2H, 2′-, 6′-H), 7.44 (d, *J* = 2.29 Hz, 1H, 5-H), 7.47 (t, *J* = 7.89 Hz, 1H, 5″-H), 8.82 (br, 1H, OH), 11.93 (br, 1H, NH); MS (ESI), *m/z* = 416 [M + H^+^]; Anal. (C_23_H_18_FN_5_O_2_) Calc. C 66.50, H 4.57, N 16.86; Found C 66.15, H 4.25, N 16.74.

*2-Amino-4-((3-chloro-4-((4-methoxybenzyl)oxy)phenyl)amino)-9H-pyrimido[4,5-b]indol-6-ol****5l*** Yield 0.111 g (5%); greyish powder; mp 141–143 °C; ^1^H NMR (DMSO-d_6_) *δ* 3.76 (s, 3H, OCH_3_), 5.21 (s, 2H, OCH_2_), 5.95 (br, 2H, NH_2_), 6.59 (br, 1H, NH-Ph), 6.63 (dd, *J* = 8.51, 2.09 Hz, 1H, 7-H), 6.86 (d, *J* = 8.51, 1H, 8-H), 6.97 (AA′**BB′**, 2H, 3″-, 5″-H), 7.39 (m, 2H, 2′-, 6′-H), 7.43 (**AA′**BB′, 2H, 2″-, 6″-H), 7.44 (s, 1H, 5-H), 7.45–7.65 (m, 1H, 5′-H), 8.85 (br, 1H, OH), 11.92 (br, 1H, NH); MS (ESI), *m/z* = 462 [M + H^+^]; Anal. (C_24_H_20_ClN_5_O_3_) Calc. C 62.41, H 4.36, N 7.67; Found C 62.25, H 4.34, N 7.45.

*4-((4–(3-Fluorobenzyl)oxy)phenyl)amino)-9H-pyrimido[4,5-b]indol-6-ol****5m*** Yield 0.041 g (2%); greyish powder; mp 190–192 °C; ^1^H NMR (DMSO-d_6_) *δ* 5.11 (s, 2H, OCH_2_), 6.32 (br, 1H, NH-Ph), 6.65 (dd, *J* = 8.76, 2.14 Hz, 1H, 7-H), 6.84 (d, *J* = 2.14, 1H, 5-H), 7.02 (AA′**BB′**, 2H, 3′-, 5′-H), 7.14 (t, *J* = 8.24 Hz, 1H, 5″-H), 7.25–7.31 (m, 2H, 2″-, 6″-H), 7.32 (**AA′**BB′, 2H, 2′-, 6′-H), 7.44 (m, 1H, 4″-H), 7.91 (d, *J* = 8.76 Hz, 1H, 8-H), 9.04 (br, 1H, OH), 9.37 (br, 1H, NH), 9.45 (s, 1H, 2-H); MS (ESI), *m/z* = 401 [M + H^+^]; Anal. (C_23_H_17_FN_4_O_2_) Calc. C 68.99, H 4.28, N 3.50; Found C 68.75, H 4.15, N 3.26.

### Receptor tyrosine kinase inhibition

The protein kinases were expressed by means of the baculovirus expression system in Sf9 insect cells as human recombinant GST fusion proteins and purified by affinity chromatography using GSH-agarose. The kinase identity was confirmed by mass spectrometry using LC-ESI-MS/MS technique.

The measuring of protein kinase activity was performed in 96-well FlashPlates^TM^ from Perkin Elmer in a 50 µL reaction volume. The reaction mixture consisted of 20 µL of assay buffer solution, 5 µL of ATP solution in water, 5 µL of used test compound in a 10% dmso solution and, finally, a premixture of each 10 µL of used substrate and enzyme solutions. The assay buffer solution contained 70 mM of HEPES-NAOH pH 7.5, each 3 mM of magnesium chloride and manganese(II) chloride, 3 µM of sodium orthovanadate, 1.2 mM of DTT, 50 µg/mL of PEG_20000_ and finally 15 µM of [γ-^33 ^P]-ATP making approximately 9 × 10^5 ^cpm per well. The final kinase concentration has been 10 ng/50 µL for EGFR and 20 ng/50 µL for PDGFR-β. The substrate was Poly(Glu,Tyr)_4:1_ for EGFR and Poly(Ala, Glu, Lys, Tyr)_6:2:5.1_ for PDFGR-β, both used in a concentration of 125 ng/50 µL. The reaction mixtures were incubated at 30 °C for 60 min. The reaction was stopped with 50 µL of a 2% (v/v) solution of phosphoric acid. Then the plates were aspirated and washed twice with 200 µL of water and a 0.9% solution of sodium chloride. The incorporation of ^33 ^P was determined with a microplate scintillation counter. Ten different inhibitor concentrations were measured in a range of 3 nM to 100 µM. The residual activity (%) and the IC_50_ values were finally calculated. From the IC_50_ values the affinity constants *K_i_* were determined using the equation: IC_50 _=1/2 [E_total_]+K_i_ × (1 + [S]/K_m_) following a competitive inhibitor binding mode^19^ (Table 1). The used K_m_ values for ATP have been measured with 1.3 µM for EGFR and with 0.989 µM for PDGFR-β.

**Table 1.** Protein kinase inhibitory activity as determined *K_i_* values of our target compounds 5a-m for the tyrosine receptor kinases EGFR and PDGFR-β.

	Ki values [nM]	
Compound	EGFR	PDGFR-β
**5a**	181 ± 14	n.a.^a^
**5b**	n.a.^a^	2750 ± 184
**5c**	149 ± 66	3530 ± 200
**5d**	935 ± 84	1910 ± 150
**5e**	574 ± 110	1944 ± 218
**5f**	421 ± 66	n.a.^a^
**5g**	686 ± 82	n.a.^a^
**5h**	475 ± 143	3230 ± 195
**5i**	413 ± 111	72 ± 15
**5j**	132 ± 99	209 ± 15
**5k**	170 ± 85	81 ± 10
**5l**	103 ± 37	213 ± 35
**5m**	189 ± 27	3660 ± 210

a not active.

### Docking studies

The 3D structures of EGFR were taken from the Protein databank. We took from the available X-ray structure the ones co-crystallised with an inhibitor structurally most similar to the pyrimido[4,5-*b*]indoles under study (PDB ID 2ITY). In addition the X-ray structure of erlotinib bound to EGFR (PDB 1M17) was taken for comparison. The protein structures were prepared as follows: water molecules and bound small molecules were deleted, hydrogen atoms were added and the resulting structures were minimised using the MMF94 force field and the conjugate gradient method until a gradient of 0.01 kcal/mol was reached. We used the docking programme GOLD 5.2 (Cambridge Crystallographic Data Centre, Cambridge, United Kingdom) to dock all compounds into the ATP binding site of EGFR. For all docking runs the programme default settings were used. The binding site was defined on the gatekeeper residue with a radius of 15 Å. Goldscore was chosen as fitness function. For each molecule, 30 docking runs were performed.

### Cellular growth inhibition assay^20^^,^^21^

Cells were inoculated into 96 well microtiter plates in 100 ml at plate densities ranging from 5000 to 40.000 cells per well depending from the individual cell doubling time. Then the cell- containing plates were incubated at 37 °C under an air atmosphere containing 5% of carbon dioxide and with a relative humidity for 24 h. After that time two plates of each cell line were fixed *in situ* with TCA as control before drug addition. Then the drug containing dmso stock solutions were used and mixed with the cell culture medium containing 50 mg/mL of gentamicin. Aliquots of 100 ml of the respective dilution were added to the preincubated plates reaching a final drug concentration of 10 µM. The plates were incubated again under the preincubation conditions as described for 48 h. Then cells were fixed under addition of 50 ml of a cold 50% solution of TCA reaching a final concentration of 10% TCA. Incubation continued for 60 min at 4 °C. The supernatant was discarded and the plates were washed with water for five times and dried at air. Then a sulforhodamine (SRB) solution (0.4%) in acetic acid (1%) was added to each well and plates were incubated again for 10 min. The unbound dye was removed by washing for five times with an acetic acid solution (1%) and the plates were dried at air. The bound stain was solubilised with a 10 µM solution of trizma base and the absorbance was measured with a plate reader at a wavelength of 515 nm. The growth inhibition measured as lowered net protein increase after SRB staining was determined in relation to the untreated control cells (Table 2).

**Table 2.** Tumour cell growth inhibition at a concentration of 10 µM of the respective inhibitors **5a**, **5k** and **5i** in screened non-small cell lung and prostate cancer cell lines.

	Compound growth inhibition [%]		
Cancer cell line	**5a**	**5k**	**5i**
Non-small cell lung cancer			
EKV X	71	90	29
NCI-H23	32	93	9
NCI-H322M	35	96	1
Prostate cancer			
PC-3	25	95	25
DU-145	51	90	15

## Results and discussion

The proceeding compound synthesis started with the formation of the 4-anilino substituted pyrimidines **2**, which were obtained by the reaction of the 4-chloro pyrimidine **1** with a set of various anilines. The alkoxy substituted anilines were prepared by alkylation of the corresponding aminophenol, using alkyl chloride and potassium tert. butoxide in DMF^22^. The ethinyl anilines were prepared using a Sonogashira reaction, from the corresponding bromo anilines dissolved in diethyl amine in the presence of methyl-3-butyn-2-ol, triphenylphosphine, copper(I) iodide, palladium(II) acetate, and, finally, with sodium hydroxide in toluene under reflux conditions^23^.

The starting compound **2** then reacted with *p*-benzoquinone in an ethanol/acetic acid mixture to give a Michael adduct **3** in the first reaction step that was further oxidised by an excess of the *p*-benzoquinone to give intermediate **4** as shown in Scheme 1. The nucleophilic amino function attack and consequent water elimination then led to our target compounds **5a–m**.

From our measured ATP competition assay with the varied target compound concentrations the EGFR affinities were calculated and presented in Table 1.

First we evaluated the various 3-anilino substituted compounds **5a**–**c**. The inhibitory activity of compound **5a** with the 3-ethoxy substituent was determined with a *K_i_* value of 181 nM. If the ethoxy substituent was replaced with a more bulky isopropoxy substituent the affinity for compound **5b** was lost. An ethinyl function placed in the 3-position of compound **5c** resulted in the best affinity with a *K_i_* value of 149 nM. So it can be stated that sterically demanding substituents in the 3-position of the aniline residue are unfavourable.

If the 3-ethoxy substituent moved to the 4-position of the aniline residue in compound **5d** the affinity towards EGFR was found decreased. If the isopropoxy substituent moved to the 4-position in derivative **5e** the affinity increased with a *K_i_* value of 574 nM. The ethinyl substituent in the 4-position also lowered the compound affinity for derivative **5f**. Halogen atoms placed in the 4-position of the aniline resulted in compound affinities of 686 nM for the 4-chloro substitution of compound **5g** and of 475 nM for the 4-bromo substitution of derivative **5h**. As indicated that more sterically demanding substituents in the 4-position of the aniline residue seem to be of little favour for the EGFR affinity we introduced benzyloxy substituents in compounds **5i**–**k**. The 4-methoxy benzyloxy substituent of derivative **5i** resulted in an affinity of 413 nM. If the 4-methoxy substituent moved to the 3-position of the benzyloxy residue the affinity increased to a value of 132 nM for compound **5j**. If replaced with a 3-fluoro substituent the affinity was almost similar with a value of 170 nM of compound **5k**. So the 3-benzyloxy substituted compounds were found as active as the 3-aniline substituted compounds. In compound **5l** we combined a 4-methoxy benzyloxy substitution with a 3-chloro aniline substitution and found the best affinity of all derivatives so far with a *K_i_* value of 103 nM. That activity almost reached the activity of erlotinib with a value of 45 nM. Finally, derivative **5m** with a 3-fluoro benzyloxy substitution and without the 2-amino function of the molecular scaffold reached a lowered affinity towards EGFR with a *K_i_* value of 189 nM.

The docking results showed that the pyrimido[4,5-*b*]indole scaffold is mimicking the adenine ring of ATP and represents the hinge-binding motifs. Potent inhibitors (e.g. **5l**) are able to form two hydrogen bonds to the hinge region residues: one to the NH of Met793 and one to the CO of Gln791. In case of compound **5m** only one hydrogen bond to the NH of Met793 is possible. The 4–(4-MeO-BnO) substituent of derivative **5l** is interacting with Lys745 as well as with hydrophobic residues at the P-loop (Phe723, Leu747, Figure 1(A)). The 3-chloro substituent is facing towards the back-hydrophobic pocket nearby Met766 that is also addressed by the cyano phenyl substituent of erlonitinb (Figure 1(B,C)).

Figure 1.(A) Docking solution for **5l** at EGFR. The inhibitor structure is coloured orange, hydrogen bonds are shown as green dashed lines. Only amino acid residues of the ATP-binding pocket are shown. (B) Visualization of the molecular surface of the ATP-binding pocket coloured according to the hydrophobicity (polar regions are coloured magenta, hydrophobic regions are coloured green). (C) X-ray structure of EGFR in complex with erlotinib (coloured green). Hydrogen bonds are shown as green dashed lines.

**Figure:**
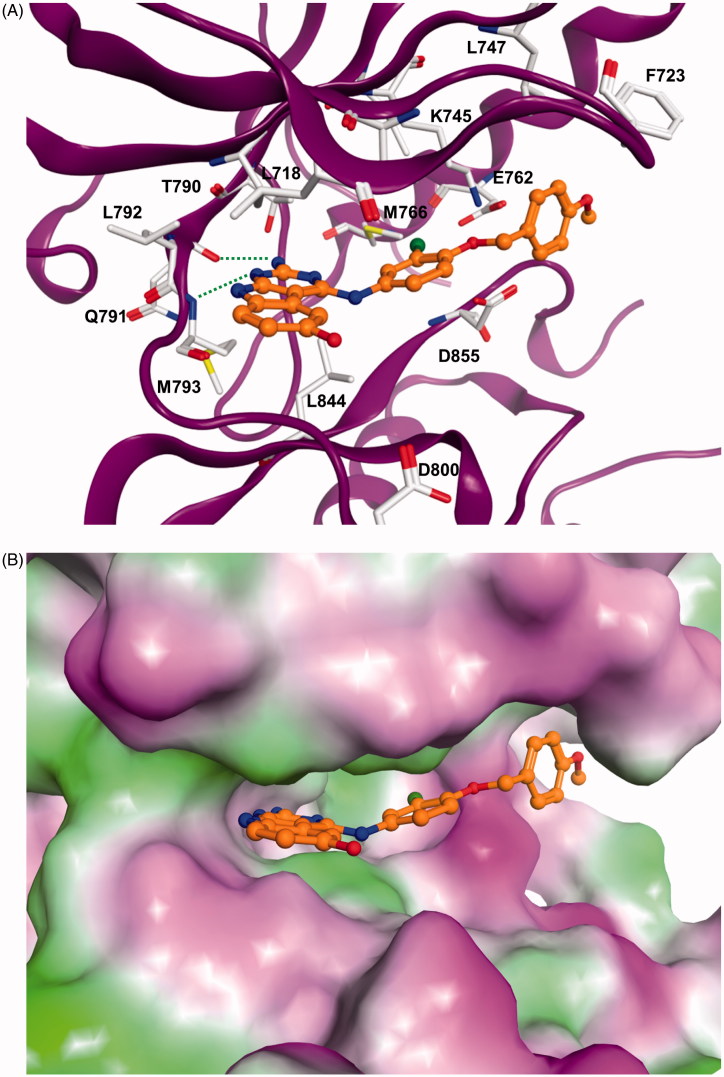

**Figure:**
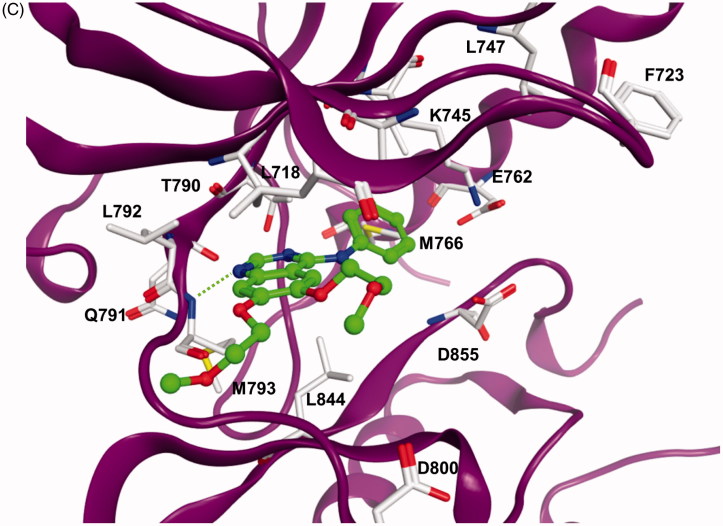

Next we evaluated our compounds to inhibit PDGFR-β in the ATP competition assay. The 3-ethoxy aniline compound **5a** was not active as PDGFR-β inhibitor. The more bulky 3-isopropoxy substituent in compound **5b** resulted in a poor affinity of 2750 nM. If replaced with the 3-ethinyl group in derivative **5c** the affinity was lowered to 3530 nM. If both alkoxy substituents were moved to the 4-position of the aniline we found improvements in the PDFG-β affinities with 1910 nM for derivative **5d** and 1944 nM for compound **5e**. The 4-ethinyl compound **5f** was not active. The smaller chloro halogen atom in the 4-aniline position was less favourable than the more bulky bromo atom which caused an inhibitory affinity of 323 nM for compound **5h**, whereas compound **5g** was not active. These first results indicated that more sterically demanding substituents in the 4-position of the aniline residue may be of favour for the PDGFR-β affinity. Indeed, we reached nanomolar PDFG-β affinities for our 4-benzyloxy substituted compounds **5i**–**k**. The 4-methoxy benzyloxy aniline substituent in compound **5i** resulted in a PDGFR-β affinity of 72 nM similar to recently reported PDFG-β inhibitors with an indolin-2-one based structure^17^. If moved to the 3-position of the benzyloxy substituent the affinity of derivative **5j** was found decreased with 209 nM. The 3-fluoro substituent, however, was more favourable in that position with an affinity constant of 81 nM for compound **5k**. If the most favourable 4-methoxybenzyloxy substitution was combined with a 3-aniline substituent we found a decrease in affinity with a value of 213 nM for derivative **5l**. Compound **5m** without the 2-amino function at the molecular scaffold showed a PDGFR-β affinity of 3660 nM which meant a decrease in the affinity by a factor of almost fifty of compared to the 2-amino compound **5k**. It can be stated that such a 2-amino function is essential for a PDFGR-β kinase affinity.

So we identified dual inhibitors of EGFR and PDFG-β and, thus, concentrated nanomolar affinities similar to sole reported EGFR and PDGFR-β inhibitors on one compound.

Next the most active compounds **5a, 5k,** and, finally, **5i** have been investigated to inhibit the growth of cancer cell types related to EGFR overexpression according to literature^4^^,^^24^. In first screening studies carried out with an initial concentration of 10 µM by the National Cancer Institute of Health (NCI) the growth inhibition of respective non-small cell lung cancer (NSCLC) cell lines and of prostate cancer cell lines was determined. The results are shown in Table 2.

The only EGFR inhibiting compound **5a** resulted in growth inhibition rates of 32 to 71% in the NSCLC cell lines and of 25 and 51% in the prostate cancer cell lines. For erlotinib for comparison growth inhibition rates of 20% in the NSCLC cell line NCI-H23 and of 40 and 45% in the prostate cancer cell lines PC-3 and DU-145 have been reported^25^^,^^26^. If the EGFR inhibition is combined with the PDGFR-β drug affinity in derivative **5k** we found main increases in the growth inhibition of the respective cell lines up to 96% in the NSCLC cell line representing NCI-H322M and of 95% in the prostate cancer cell line PC-3. Compound **5i** with a similar PDGFR-β affinity than derivative **5k**, but a reduced EGFR affinity, also lower than that of compound **5a**, resulted in a reduced growth inhibition with rates ranging from just 1 to 29% in the NSCLC cell lines and of 15 to 25% in the prostate cancer cell lines. These results reveal the importance of an EGFR inhibition in anticancer cell line growth and prove a certain potency of that inhibition with a combined inhibition of PDGFR-β.

It can be shortly summarised for our novel compound class that large substituents in the 4-position of the aniline residue favour both the inhibition of EGFR and PDGFR-β. The dual inhibitors with favourable EGFR-affinities show an effective anticancer growth as far as investigated. So they may be of benefit to combat an anticancer drug resistance resulting from a receptor heterodimerization as suggested by our first results.
