# Correction to: Fischer et al., Discovery of novel dual inhibitors of receptor tyrosine kinases EGFR and PDGFR-β related to anticancer drug resistance

**DOI:** 10.1080/14756366.2018.1433261

**Published:** 2018-02-02

Fischer T, Najjar A, Totzke F, Schächtele C, Sippl W, Ritter C and Hilgeroth A. (2017). Discovery of novel dual inhibitors of receptor tyrosine kinases EGFR and PDGFR-β related to anticancer drug resistance. *Journal of Enzyme Inhibition and Medicinal Chemistry*. 33:1–8. https://doi.org/10.1080/14756366.2017.1370583.

When the article was first published online, the name of one co-author was incorrectly written as Karim Najjar. This has now been corrected in the online version as ‘Abdulkarim Najjar’.

The authors apologise for this error.

